# Advancing Obstructive Sleep Apnea Management: Recent Trends from Conventional to Innovative Therapies

**DOI:** 10.3390/jcm14217586

**Published:** 2025-10-26

**Authors:** Soo Kyoung Park, Ji Ho Choi

**Affiliations:** 1 Department of Otorhinolaryngology-Head and Neck Surgery, Chungnam National University Sejong Hospital, College of Medicine, Chungnam National University, Daejeon 34134, Republic of Korea; pacsoo2@cnuh.co.kr; 2 Department of Otorhinolaryngology-Head and Neck Surgery, Soonchunhyang University Bucheon Hospital, College of Medicine, Soonchunhyang University, 170, Jomaru-ro, Bucheon 14584, Republic of Korea

**Keywords:** sleep apnea, obstructive, positive airway pressure, patient compliance, hypoglossal nerve, drug therapy

## Abstract

Obstructive sleep apnea (OSA) is a common disorder characterized by recurrent upper airway collapse during sleep, leading to intermittent hypoxemia and sleep fragmentation. Untreated OSA is associated with increased risks of cardiovascular, metabolic, and neurocognitive comorbidities, as well as considerable socioeconomic burden. Positive airway pressure (PAP) remains the gold standard therapy; however, limited long-term adherence underscores the need for alternative, patient-centered approaches. Conventional modalities such as oral appliances, surgery, weight reduction, and positional therapy provide clinical benefits but have variable efficacy and tolerability. Recent advances highlight innovative strategies, including hypoglossal nerve stimulation (HGNS), anti-obesity pharmacotherapy with glucagon-like peptide-1 receptor agonists, and upper airway muscle–targeted agents, which exemplify precision medicine approaches tailored to individual OSA phenotypes. This review synthesizes current evidence on both conventional and emerging therapies, emphasizing the transition from a “one-size-fits-all” model toward integrated, phenotype-driven management aimed at improving outcomes and quality of life for patients with OSA.

## 1. Introduction

Obstructive sleep apnea (OSA) is a disorder characterized by the partial (hypopnea) or complete (apnea) cessation of airflow during sleep due to recurrent upper airway collapse. This collapse commonly arises from diminished pharyngeal muscle tone and structural abnormalities, leading to intermittent hypoxemia and repeated sleep fragmentation [1,2]. In contrast to simple snoring, OSA induces significant systemic effects: recurring episodes of apnea prompt sympathetic nervous system activation, elevate blood pressure, and increase cardiac workload, thereby promoting a wide spectrum of adverse health outcomes [3,4,5].

Population-based studies indicate that the prevalence of moderate OSA (AHI ≥ 15 events/h) is approximately 13% in men and 6% in women, whereas mild OSA (AHI ≥ 5) may affect up to 26% of men and 9% of women, consistent with previously reported epidemiologic estimates [6,7]. Major risk factors encompass obesity, advancing age, male sex, family history, and anatomical variations of the upper airway [8]. Recent research has emphasized the clinical significance of OSA beyond obesity, with increasing recognition of diverse phenotypes—including those in children, women, and non-obese individuals—highlighting the heterogeneous nature of the disorder [9]. Nonetheless, a substantial proportion of individuals remain undiagnosed or untreated, representing a significant public health issue [10]. The socioeconomic impact of OSA—including escalating healthcare costs, diminished workplace productivity, and increased incidence of traffic accidents—is considerable, and diagnostic or therapeutic delays further intensify these consequences [11].

OSA is not solely a sleep disorder; it also serves as a significant risk factor for, or exacerbates, numerous chronic diseases. Recurrent episodes of apnea and the associated hypoxemia increase the risk of cardiovascular conditions (such as hypertension, heart failure, myocardial infarction, and stroke) as well as metabolic disorders (including insulin resistance and type 2 diabetes) [9]. OSA is further linked to neurocognitive and psychological disturbances, manifesting as inattention, impaired memory, depression, and anxiety [12,13]. In men, declines in testosterone concentrations resulting in sexual dysfunction have been documented, and associations with gastroesophageal reflux disease as well as nocturnal enuresis in both adults and children have been reported [14,15,16,17]. Thus, absent prompt diagnosis and effective intervention, OSA can contribute to progressive deterioration of both physical and mental health over time.

A variety of therapeutic modalities—including positive airway pressure (PAP), oral appliances, surgery, weight management, and positional therapy—are utilized in the management of OSA [18]. Among these, PAP is the most commonly prescribed worldwide and is recognized for its high efficacy; nevertheless, its effectiveness in clinical settings is frequently compromised by inadequate adherence and patient discomfort [19]. Patient-specific factors such as anatomical differences, physiological characteristics, and comorbidities vary greatly and are often insufficiently addressed by standardized PAP therapy, resulting in less than optimal therapeutic outcomes. As a result, there has been increasing attention to individualized treatment approaches that emphasize non-invasive options, promote durable adherence, and incorporate novel therapies to supplement or replace traditional interventions [20,21].

This review examines both established therapies and recently developed, innovative modalities that are receiving increasing focus in contemporary research. Specifically, we highlight hypoglossal nerve stimulation (HGNS), anti-obesity pharmacotherapy, and agents that modulate upper-airway muscle tone, while also addressing future trends in OSA management. Our objective is to support the development of precision-based, patient-centered care pathways that enhance OSA management in clinical settings and facilitate individualized therapeutic planning.

## 2. Conventional Therapies

### 2.1. PAP

PAP therapy ensures airway patency during sleep by delivering pressurized air to physically prevent upper airway narrowing or closure. The device operates by drawing in room air with a pump, filtering it, and then delivering the air at a specified pressure through tubing and a mask directly to the upper airway (including the nasal cavity, pharynx, and larynx). The positive pressure opposes the inspiratory negative pressure that can lead to airway collapse, thereby maintaining airway patency [22]. By keeping the airway open, normal airflow to the lungs is maintained, preventing apnea, hypopnea, oxygen desaturation, and arousals related to breathing disturbances—consequently improving sleep quality and reducing daytime symptoms [19].

High-level evidence from multiple randomized controlled trials shows that PAP can lower the apnea–hypopnea index (AHI) to less than 10 events per hour in a significant proportion of patients, while also improving both subjective and objective measures of daytime sleepiness [23]. PAP therapy further improves quality of life, enhances sleep consolidation by increasing N3 sleep, and reduces both nocturnal and daytime systemic blood pressure [24,25,26]. Additional systemic benefits reported include a reduction in cardiovascular risk [27], decreased risk of motor-vehicle crashes [28], improvement in nocturia [29], and decrease in heightened sympathetic activity [3]. Favorable changes have also been observed in inflammatory and microvascular markers—such as reductions in fibrinogen, C-reactive protein, interleukin-6, vascular endothelial growth factor, and platelet activation/aggregation [30,31,32]. For patients with heart failure or atrial fibrillation, PAP has been shown to improve ejection fraction and decrease the recurrence of arrhythmias [33]. Of note, snoring typically decreases, which leads to improvements in sleep quality for both patients and their bed partners [34].

Although PAP is effective, its limitations frequently result in less than optimal long-term adherence [35]. Some studies have reported adherence rates as low as 29% [19,36]. Frequently cited obstacles include issues related to the mask—such as pressure marks, erythema, irritation, even ulceration; air leaks that diminish therapeutic effectiveness and cause ocular dryness; and feelings of claustrophobia or discomfort in certain individuals [37,38]. Pressure-associated adverse effects should also be considered, including nasal and pharyngeal dryness that may result in congestion, thirst, or epistaxis; aerophagia with accompanying bloating or gas; and difficulty exhaling against the prescribed airflow in some users [39,40]. In addition, noise from the device can disrupt sleep for both patients and partners, and the need for portability during travel, as well as regular cleaning and maintenance of masks and tubing, introduces additional practical challenges [41,42,43]. Importantly, while PAP effectively addresses upper-airway narrowing, it does not resolve the fundamental anatomical or physiological contributors to OSA, and therefore should not be considered curative [44].

### 2.2. Oral Appliance

Oral appliance therapy, most commonly utilizing a mandibular advancement device (MAD), acts by physically altering the upper airway structure to maintain airway patency. The MAD is custom-fitted to both the maxillary and mandibular dentition and is specifically designed to advance the mandible slightly during sleep while limiting backward displacement and lateral movement. These devices are individualized to accommodate each patient’s unique dental and mandibular configuration [45,46]. Mandibular advancement simultaneously draws the tongue base and soft palate (including the uvula) forward, thereby mechanically preventing these structures from collapsing posteriorly under gravitational forces during sleep and enlarging the upper airway lumen [45,47]. Several studies additionally indicate that MAD application enhances upper airway structural stability by anteriorly repositioning surrounding soft tissue [48]. Following airway enlargement and stabilization, air passage to the lungs is improved, leading to reductions in apnea, hypopnea, and snoring, as well as preservation of oxygen saturation (SpO_2_) and decreased sleep fragmentation—ultimately contributing to improved sleep quality overall [49]. Most MADs are designed to be titratable; mandibular advancement begins in small, controlled increments and is gradually adjusted to achieve optimal clinical benefit [50].

Oral appliances demonstrate particular efficacy in individuals with mild-to-moderate OSA or in those unable to tolerate PAP therapy [50]. MADs have been shown to significantly decrease AHI and to ameliorate core symptoms of OSA, such as snoring and excessive daytime sleepiness. Notably, evidence from several randomized and comparative studies indicates that MADs can provide symptomatic improvement comparable to PAP therapy in mild-to-moderate OSA cases [51]. Compared with PAP devices, oral appliances are less bulky, more portable, and quieter, which can lead to higher patient adherence [45]. This compliance advantage is important for sustained long-term use. Symptomatic improvements in daytime sleepiness and sleep quality are associated with reduced fatigue, enhanced mood, improved cognitive performance, and overall better quality of life [52]. Further, some publications suggest that MAD therapy may have beneficial effects on cardiovascular risk factors, including hypertension [53,54].

Despite their benefits, oral appliances have notable limitations. Their therapeutic effectiveness differs significantly among individuals, and accurately identifying likely responders before treatment initiation remains challenging [55]. Monotherapy with MAD is often insufficient in cases of severe OSA [56]. Sustained mandibular advancement can exert pressure on the temporomandibular joint, potentially causing pain, joint sounds, or functional limitations; prolonged use may also lead to minor tooth shifts or changes in dental occlusion [57,58,59]. Therefore, it is important to conduct periodic dental assessments and ongoing monitoring. Patients may experience changes in saliva production, such as excessive salivation or dryness, as well as gum or tooth irritation and local discomfort during the early adaptation phase [60]. Consistent nightly use of oral appliances is required—similar to PAP—to maintain therapeutic benefit. Long-term adherence rates have been reported at about 6% to 70%, with discontinuations primarily due to discomfort, inadequate effectiveness, or dental adverse effects [61,62,63]. Fabrication of these devices is individualized, necessitating involvement by a specialist, while proper hygiene and device upkeep must also be ensured [64]. Finally, while oral appliances can effectively reduce symptoms by enlarging the airway, they do not address the fundamental anatomical or physiological mechanisms of OSA, and thus do not provide a definitive cure [60].

### 2.3. Surgery

Surgical intervention for OSA aims to address structural causes of upper airway obstruction by targeting sites including the nasal cavity, soft palate, lateral pharyngeal wall, tongue base, and mandible. Each surgical modality focuses on augmenting or stabilizing anatomical regions to maintain upper airway openness [65,66].

Uvulopalatopharyngoplasty (UPPP) remains one of the most widely used surgical treatments for OSA. The procedure excises redundant tissue from the soft palate, uvula, tonsils, and pharyngeal walls in order to expand the retropalatal airway. UPPP is generally most effective in individuals whose obstruction is mainly attributable to an elongated soft palate or enlarged tonsils [65,67].

Tongue base reduction is indicated in cases where airway obstruction is caused by an enlarged or posteriorly displaced tongue base. The intervention decreases tongue base mass using techniques such as radiofrequency ablation, laser therapy, or partial surgical removal, thereby increasing pharyngeal space. Recently, minimally invasive approaches such as transoral robotic surgery and coblation-assisted procedures have become increasingly common [68,69,70].

Septoplasty and turbinate reduction are performed to correct nasal anatomical issues—including septal deviation or turbinate hypertrophy—that result in nasal blockage. By decreasing upper airway resistance and reducing the need for mouth breathing, these procedures can lessen the risk of airway collapse during sleep [71,72,73,74].

Maxillomandibular advancement (MMA) is recognized as the most effective surgical intervention for OSA. By advancing both the maxilla and mandible, this surgery increases craniofacial skeletal volume, resulting in anterior displacement of the tongue, soft palate, and associated soft tissues, thereby enlarging the upper airway. MMA shows marked efficacy in moderate-to-severe OSA or in patients who have not benefited from other surgical interventions, with literature reporting AHI reductions as high as 87% [75,76].

Surgical therapy has been demonstrated to improve OSA symptoms and respiratory parameters in appropriately selected individuals. In addition to lowering AHI, surgical treatment can reduce snoring, daytime sleepiness, and morning headaches, and is associated with enhanced quality of life, sleep satisfaction, mood, and cognitive function. Among the surgical approaches, MMA achieves the highest rates of success and sustained clinical benefit, representing a critical option for patients who cannot tolerate or decline PAP therapy [66,77].

Notwithstanding these advantages, surgical management also involves significant limitations and risks. Effectiveness differs based on procedure type, anatomical characteristics, and disease severity. MMA is associated with a high rate of success and is widely accepted as an effective option for moderate-to-severe OSA [65,77], while single-site interventions such as UPPP yield lower success rates (30–50%) and variable outcomes [78,79,80]. As an invasive intervention, surgery carries potential complications such as bleeding, infection, edema, pain, sensory alterations, voice changes, and dysphagia. Recovery after MMA often requires an extended period and considerable patient commitment [65,81,82]. Long-term recurrence can develop, particularly due to weight gain or anatomical shifts, and thus prolonged surveillance is recommended [83]. Therefore, surgical intervention should primarily be reserved for individuals with well-identified obstructive locations; for those with possible multilevel airway obstruction, consideration should be given to multistage surgical plans or adjunctive nonsurgical treatments [65,84,85].

### 2.4. Weight Loss

Obesity represents one of the strongest risk factors for OSA. The deposition of adipose tissue in the neck and abdomen enlarges the volume of parapharyngeal structures, thereby increasing susceptibility to upper airway narrowing or collapse during sleep. Additionally, abdominal obesity raises the diaphragm, diminishes lung volumes, and thereby promotes further upper airway instability [86]. Weight reduction lowers adipose tissue surrounding the airway and enhances airway patency, and this mechanism underlies the effectiveness of weight loss as a therapy for OSA [87,88,89,90].

Weight loss can reduce OSA severity and, in certain cases, may lead to near-complete resolution of the disorder. A study demonstrated that a 10% decrease in body weight was associated with an average 26% reduction in AHI, whereas a 10% increase in weight was linked to an approximately 32% rise in AHI [91]. These results quantitatively highlight the direct influence of weight fluctuations on OSA severity. Furthermore, weight loss ameliorates common OSA comorbidities—such as hypertension, type 2 diabetes, dyslipidemia, and cardiovascular disease—thus offering wider health benefits and enhancing sleep quality [92,93,94].

However, maintaining long-term weight loss remains a significant challenge. Sustained success requires comprehensive lifestyle changes and high motivation, yet many patients struggle to adhere to these modifications, thereby limiting long-term effectiveness [95,96]. In addition, since weight reduction is a gradual process, immediate improvements in symptoms should not be expected, and for individuals with moderate-to-severe OSA, relying solely on weight loss often does not provide sufficient therapeutic effect [88]. Consequently, weight reduction should be implemented in conjunction with other treatments such as continuous positive airway pressure (CPAP) to maximize therapeutic outcomes, emphasizing the importance of a comprehensive treatment strategy [97].

### 2.5. Positional Therapy

Body position during sleep has a substantial effect on both the frequency and severity of OSA [98]. Supine sleep allows gravitational forces to shift the tongue and soft palate backward, which narrows or blocks the upper airway. Alternatively, lateral or prone positions are linked to a decreased probability of airway collapse [98,99,100]. If the AHI while supine is more than double that of the non-supine position, the condition is defined as “positional OSA” [101]. The aim of positional therapy is to prevent supine sleep and promote consistent lateral or prone positioning, which lessens gravitational impact and helps maintain airway patency [102].

Positional therapy is especially beneficial for individuals with mild-to-moderate positional OSA, characterized by significantly higher AHIs when supine but exhibiting normalization or major reduction in non-supine positions. By reducing time spent sleeping in the supine position, positional therapy lowers AHI and improves other respiratory parameters, such as minimum SpO_2_ [103]. Recent meta-analytic evidence showed that positional therapy significantly enhances respiratory outcomes in patients with positional OSA, resulting in both AHI reduction and increased SpO_2_ [104]. These findings highlight its role as an effective non-invasive intervention that delivers clinically meaningful benefits to carefully selected patients.

However, positional therapy is not suitable for every patient. For individuals whose apneas are not related to sleep position, the efficacy of this intervention is very limited. In cases of severe OSA, positional therapy as a sole treatment is often inadequate because it fails to properly maintain airway patency [105]. Additionally, many patients involuntarily shift their position during sleep, making it challenging to uphold a stable lateral or prone posture throughout the night [106]. Moreover, the devices employed to facilitate positional therapy can cause discomfort or disturb sleep, creating further barriers to sustained adherence [107].

### 2.6. Orofacial Myofunctional Therapy (OMT)

Orofacial Myofunctional Therapy (OMT) retrains tongue and peri-pharyngeal muscle function to reduce upper-airway collapsibility. Several systematic reviews and meta-analyses have confirmed that OMT produces modest but statistically significant reductions in apnea–hypopnea index (AHI) and Epworth Sleepiness Scale (ESS) scores, particularly when used as an adjunct to CPAP or mandibular advancement devices [108,109,110].

Furthermore, a recent clinical study reported that OMT as an adjunctive intervention improved auto-PAP adherence and reduced required therapeutic pressure levels after three months of therapy [111]. However, patient adherence remains a major challenge; mobile-health–based training and remote supervision approaches have shown potential to improve compliance and engagement in preliminary studies [112].

## 3. Innovative Therapies

### 3.1. HGNS

HGNS, also referred to as upper airway stimulation (UAS), is an implantable therapeutic approach designed to prevent upper airway collapse during sleep. The system operates by delivering respiration-synchronized electrical stimulation to the hypoglossal nerve, which innervates the genioglossus and geniohyoid muscles [113,114]. As a result, this stimulation moves the tongue forward and increases its stiffness, thereby enhancing airflow, preserving pharyngeal patency, and lowering or eliminating airway obstruction [113,115].

HGNS is principally indicated for adults (≥18 years) with moderate-to-severe OSA who are intolerant of, or unwilling to use, CPAP [113,114]. According to AASM guidelines and FDA labeling, poor compliance with CPAP is defined as average use <4 h per night or use on <70% of nights, or clinical intolerance preventing continued use, which constitutes a principal indication for consideration of HGNS [113,114]. Typically, eligible patients have an AHI between 15 and 65 events/h (20–50 events/h in certain studies). The sleep-disordered breathing pattern must be predominantly obstructive, and the proportion of central or mixed apneas should constitute <25% of total AHI [113,116]. Documented CPAP intolerance or failure—defined as persistently poor adherence even after comprehensive counseling and optimization—is a prerequisite [113]. Most HGNS systems have a body mass index (BMI) requirement of ≤32 kg/m^2^ based on pivotal trial criteria [113], though some devices allow use in individuals up to ≤35 kg/m^2^ [117]. Recent FDA updates and registry data have further expanded eligibility to patients with BMI up to ≤40 kg/m^2^ [118]. Assessment of anatomical factors is essential, and drug-induced sleep endoscopy (DISE) is considered mandatory for selecting appropriate candidates. In particular, complete concentric collapse (CCC) at the velum serves as an exclusion criterion for some systems, such as Inspire, which makes DISE a critical part of pre-implantation evaluation [116,119]. These rigorous selection criteria are intended to improve outcome success rates and optimize clinical results [113,116]. Patients with neuromuscular disease, hypoglossal nerve palsy, clinically significant restrictive or obstructive lung disease, moderate-to-severe pulmonary hypertension, severe valvular heart disease, heart failure (NYHA class III–IV), myocardial infarction or unstable arrhythmia within the previous 6 months, uncontrolled hypertension, severe psychiatric illness, other coexisting sleep disorders, or anatomical findings such as tonsil size 3 to 4 are generally excluded from candidacy [113].

Inspire Medical Systems (Inspire^®^; Inspire Medical Systems, Inc., Maple Grove, MN, USA) developed the first U.S. Food and Drug Administration (FDA)-approved UAS device. This system comprises three implantable components: a subclavicular pulse generator, a chest wall sensing lead designed to detect respiratory effort, and a stimulation cuff electrode encircling a hypoglossal nerve branch. Earlier HGNS models utilized a separate chest sensing lead; however, the latest Generation V device integrates the sensor within the pulse generator, simplifying implantation and reducing lead-related complications [118]. Recent FDA clearance has allowed next generation Inspire V devices to enter clinical use, with preliminary reports showing improved procedural efficiency and comparable therapeutic outcomes [120]. Device activation and parameter adjustment are performed by the patient using an external remote control. The Inspire system provides unilateral and respiration-synchronized stimulation [113]. In the STAR trial, selected patients achieved a median reduction in AHI of 68% (from 29.3 to 9.0 events/h), with 66% of patients attaining both ≥50% AHI reduction and an AHI < 20 events/h [121]. Five-year follow-up demonstrated a surgical success rate of 75%, and reported median decreases of 67.4% in AHI and 67.5% in oxygen desaturation index (ODI), as well as marked improvements in patient-reported outcomes, such as quality of life (Functional Outcomes in Sleep Questionnaire [FOSQ]) and daytime sleepiness (Epworth Sleepiness Scale [ESS]) [122]. The safety profile is considered acceptable, with most adverse events being non-serious, including tongue soreness, incisional pain, and transient tongue weakness; approximately 6% of patients required device reprogramming or additional intervention [122]. Battery life typically ranges from 8 to 12 years, after which replacement surgery is needed [113]. The estimated total cost, including device and surgical implantation, is $30,000 to 40,000 prior to insurance reimbursement [123]. Despite the high initial cost, long-term economic benefits are anticipated due to the prevention of complications arising from untreated OSA.

LivaNova’s aura6000™ system utilizes proximal HGNS. In contrast to Inspire, which delivers stimulation to distal hypoglossal nerve branches, the aura6000 targets the proximal trunk through six electrodes, activating a broader group of airway control muscles and allowing for individualized threshold adjustment [124,125]. Schwartz et al. [124] reported that treatment with aura6000 resulted in AHI and ODI response rates of 52.3% and 62.5%, respectively, at 4 months, compared with 19.6% and 41.3% in the sham group; these improvements were maintained at 12–15 months. Clinically meaningful improvements were noted in ESS, FOSQ, and EQ-5D, and serious device-related adverse events included one case of lead dislodgement and one case of local pain [124]. Additional studies underscore advantages such as a more streamlined surgical approach, the potential to eliminate the need for DISE, and fewer incisions, which together may improve patient experience compared with previous technologies [125]. The aura6000 has been granted FDA Investigational Device Exemption and is currently being evaluated in the pivotal OSPREY trial (NCT05050400), which aims to assess long-term safety and efficacy in moderate-to-severe OSA [124,125].

Nyxoah’s Genio™ system presents a novel bilateral stimulation method. The device utilizes compact, wireless, battery-free implantable electrodes that are activated nocturnally by an external patch and chip placed beneath the chin. This configuration permits a single submental incision, maintains magnetic resonance imaging (MRI) compatibility, and supports convenient adjustments or upgrades without further surgical intervention [126]. Initial clinical studies have reported marked improvements in AHI and ODI at the 6-month mark, coupled with high levels of patient satisfaction regarding sleep quality and daytime alertness, without major device-related adverse events [126]. In 2024, details of the DREAM pivotal study protocol were released, describing a multicenter, prospective, open-label investigation involving 10 clinical sites across the United States, Europe, and Australia, recruiting approximately 115 adults with moderate-to-severe OSA. Primary outcomes focus on shifts in AHI and ODI, while secondary endpoints include patient-reported outcomes and monitoring of adverse events [127,128]. Most recently, the Genio system was granted FDA approval in 2025, and its procedural cost is expected to match that of Inspire (roughly $40,000). Relative to Inspire, Genio delivers distinct benefits such as a streamlined procedure, decreased invasiveness, improved convenience for patients, and selection without DISE, which could extend the clinical utility of HGNS [126].

Despite HGNS emerging as a significant advancement for OSA management, it retains several limitations. The intervention necessitates invasive surgery, bearing risks like bleeding, infection, postoperative pain, and anesthesia-related complications [129]. Strict eligibility criteria—including BMI thresholds and CCC presence at the velum—also restrict patient access [114]. The financial burden of devices and surgical intervention is considerable, with the implanted battery generally requiring replacement after 8 to 12 years [113]. Additional drawbacks include postoperative recuperation, delayed achievement of optimal therapeutic effect due to gradual titration, and the potential for side effects such as lingual discomfort, abrasion, changes in tongue motility, device failure, or uncommon events like nerve injury [129]. Additionally, HGNS is designed to target specific upper airway obstruction sites and may be insufficient in patients with complex or multifactorial etiologies [114]. MRI compatibility can differ between devices, and long-term evidence for both effectiveness and safety is still evolving [129] (Table 1).

### 3.2. Anti-Obesity Medication (GLP-1 Receptor Agonists)

Glucagon-like peptide-1 receptor agonists (GLP-1 RAs) constitute a class of medications that emulate the physiological effects of endogenous GLP-1, which is released postprandially in the gastrointestinal tract; these agents facilitate appetite suppression, delay gastric emptying, and improve glucose regulation [130]. Notably, GLP-1 RAs target specific receptors in the central nervous system (e.g., hypothalamus) to promote satiety and decrease appetite, resulting in lower total energy consumption [130,131]. They additionally retard gastric emptying, thereby sustaining postprandial satiety and further decreasing caloric intake [131]. Furthermore, GLP-1 RAs enhance insulin secretion and inhibit glucagon production in a glucose-dependent fashion, effectively lowering blood glucose levels and promoting insulin sensitivity [130,132].

GLP-1 RAs demonstrate substantial efficacy in inducing clinically significant and durable weight loss. As an example, tirzepatide achieved an average body-weight reduction of 17.7% in the PAP-non-use group and 19.6% in the PAP-use group over the 52-week randomized SURMOUNT-OSA study in individuals with obesity and moderate-to-severe OSA [133]. Weight loss is regarded as the principal mechanism by which GLP-1 RAs ameliorate OSA severity, primarily because obesity is a major risk factor and parapharyngeal adiposity increases airway collapsibility. The same trial established a strong relationship between weight reduction and decreases in AHI [133]. Marked fat loss often occurs within 3 months after initiation, promoting the rapid improvement of obesity-related conditions such as OSA. A recent meta-analysis indicated that GLP-1 RAs reduced AHI by an average of −9.48 events/hour, with tirzepatide yielding greater effects compared to liraglutide [134].

Considering the strong interplay among OSA, obesity, and type 2 diabetes [87], GLP-1 RAs offer several clinical advantages. Aside from facilitating weight reduction and improving OSA, they also enhance cardio-metabolic outcomes such as glycemic management and blood pressure control [135,136]. Consequently, GLP-1 RAs not only target obesity—the primary pathophysiologic substrate of OSA—but also provide extensive metabolic benefits, functioning as a pharmacological intervention that connects these interrelated disease processes [137].

Tirzepatide (Zepbound™) is a dual GIP/GLP-1 receptor agonist administered once weekly via subcutaneous injection. It is approved by the FDA for type 2 diabetes (Mounjaro) and for obesity/OSA (Zepbound™). In certain clinical trials, tirzepatide produced greater weight loss than semaglutide, with mean reductions of 20.2% compared to 13.7%, respectively [138]. In studies involving OSA, tirzepatide led to substantial body weight reductions (17.7–19.6%) and pronounced decreases in AHI (−20 to −23.8 events/hour) among obese patients with moderate-to-severe OSA [133]. Similar to other GLP-1 RAs, the most frequent side effects are gastrointestinal in nature (including nausea, vomiting, diarrhea, abdominal pain, and constipation). Uncommon but serious adverse effects encompass pancreatitis, gallbladder pathology, acute kidney injury, hypoglycemia, severe hypersensitivity reactions, and a potential risk for thyroid C-cell neoplasia [139,140].

Semaglutide (Ozempic^®^, Wegovy^®^, Rybelsus^®^) is formulated as a once-weekly subcutaneous injection (Ozempic^®^, Wegovy^®^) or a daily oral tablet (Rybelsus^®^). Wegovy^®^ holds FDA approval for obesity, whereas Ozempic^®^ and Rybelsus^®^ are approved for type 2 diabetes. Clinical studies have consistently shown significant weight reduction with semaglutide 2.4 mg (average 12.4%, with individual responses approaching ~20%) [141]. Despite these results, there are currently no prospective trials evaluating semaglutide’s direct effects on OSA. The adverse event profile is consistent with other GLP-1 RAs, primarily involving gastrointestinal symptoms that are generally mild and transient. Rare but clinically relevant adverse events include pancreatitis, gallbladder disease, renal impairment, elevated heart rate, and contraindication in those with a personal or family history of medullary thyroid carcinoma or MEN2 [141,142].

Liraglutide (Saxenda^®^) is administered as a daily subcutaneous injection. The high-dose formulation (3.0 mg) is FDA-approved for long-term weight management in adults with obesity or overweight [143]. Data support liraglutide’s efficacy in improving OSA severity alongside weight loss, with systematic reviews and meta-analyses demonstrating significant reductions in AHI (−5.69 events/hour) [134,144]. Its adverse effects are similar to other GLP-1 RAs, mostly gastrointestinal symptoms (nausea, vomiting, diarrhea, constipation), typically subsiding within several weeks after treatment begins [143,144].

GLP-1 RAs were first approved for type 2 diabetes and weight management, but the FDA has recently added an indication for tirzepatide (Zepbound™) for treatment of OSA associated with obesity [133]. This expansion reflects ongoing advancements toward pharmacological therapies that may reduce dependence on device-based treatments such as CPAP, oral appliances, or surgery, particularly for OSA associated with obesity.

Despite their benefits, GLP-1 RAs present certain limitations. Gastrointestinal side effects remain the most frequent (nausea, vomiting, diarrhea, constipation, abdominal discomfort), and some patients may discontinue therapy due to intolerability [145]. Infrequently, pancreatitis, gallbladder disease, renal impairment, and thyroid C-cell tumors can occur, requiring vigilant clinical monitoring [146]. Cost—especially for uninsured patients—is a considerable barrier to widespread access [145]. Additionally, loss of lean body mass, particularly skeletal muscle, may accompany weight loss and should be monitored [147,148]. Finally, the greatest efficacy is observed in obesity-related OSA, and GLP-1 RAs are not effective for all pathophysiological subtypes of the disease. In cases where non-obesity anatomical factors are predominant, GLP-1 RAs may be best utilized as adjunctive therapy [149,150] (Table 2).

### 3.3. Upper Airway Muscle Modulating Agents

Pharmacologic treatment options for OSA were historically limited, but recent advancements have introduced novel drugs that act directly on the underlying pathophysiological mechanisms of OSA, independent of obesity. This marks a significant progression toward precision medicine strategies that incorporate the diverse pathophysiology of OSA [21,151].

AD109, being developed by Apnimed, is an oral fixed-dose combination specifically formulated to target neuromuscular impairment responsible for upper airway collapse—the central pathophysiologic defect in OSA. AD109 contains atomoxetine and aroxybutynin. Atomoxetine, a selective norepinephrine reuptake inhibitor, increases the sympathetic activity of the hypoglossal motor nucleus, leading to increased tone of the upper airway musculature during sleep. Earlier proof-of-concept studies using a prototype combination of atomoxetine and oxybutynin demonstrated increased upper-airway muscle tone and reduced pharyngeal relaxation during sleep [152,153]. Building on these findings, the final fixed-dose formulation AD109 (atomoxetine + aroxybutynin) was optimized to provide synergistic enhancement of upper-airway dilator muscle activity across sleep stages, resulting in significant reductions in apnea and hypopnea events in clinical trials [154].

Among these, the MARIPOSA trial evaluated repeated nightly dosing of AD109 and reported approximately 45% reductions in AHI, together with significant improvements in ODI, hypoxic burden, and arousal index, rather than >50% reductions after a single dose [154]. Importantly, these benefits occurred after only a single dose, confirming the drug’s rapid onset of action and highlighting its capacity to target key disease mechanisms [153]. Research by Taranto-Montemurro and colleagues [155] also revealed that response to the therapy differed by patient endotype, with clear physiologic effects across various core OSA traits such as pharyngeal collapsibility, arousal threshold, loop gain, and muscle responsiveness. They found that the atomoxetine/aroxybutynin combination delivered improvements in both muscle responsiveness and arousal threshold simultaneously. These results indicate that pharmacologic therapy can address multiple factors involved in the pathogenesis of OSA, further lending support to precision medicine–oriented approaches in drug development for OSA [152,155].

Additionally, Taranto-Montemurro et al. [152] highlighted the practical advantages of this oral drug combination, including its non-invasive route and the likelihood of improved patient compliance compared to CPAP, suggesting its value for chronic management. Clinical studies have shown generally favorable tolerability of the therapy; most adverse events were mild (such as dry mouth or headache), with serious adverse events occurring infrequently [153,154].

Because AD109 primarily exerts its effect through neuromodulation of upper airway muscle activity, patients with severe anatomical obstruction (e.g., markedly enlarged tonsils, macroglossia) may not achieve adequate efficacy with monotherapy [152,154]. Additionally, interindividual differences in drug responsiveness complicate the ability to ensure consistent efficacy among all patients, and long-term data on safety and effectiveness remain insufficient. Notably, the potential risks linked to chronic multi-year use are not yet fully understood and warrant further study [152,155]. Since AD109 has mostly been evaluated in patients who are intolerant of or decline CPAP, its relative benefit in individuals who tolerate CPAP remains undetermined. Moreover, specific patient populations—such as those with concomitant central sleep apnea or particular underlying comorbidities—may not be suitable for this therapy, emphasizing the necessity for careful patient selection.

## 4. Conclusions

Management of OSA has progressed from early invasive surgical techniques to current standards that prioritize PAP and oral appliance therapy, and is now transitioning toward advanced, precision medicine strategies that integrate endotype- and phenotype-driven approaches such as HGNS, anti-obesity drug therapy, and agents that enhance upper airway muscle tone. While PAP remains the gold standard for moderate-to-severe OSA, suboptimal patient adherence continues to be a significant barrier. Oral appliance therapy is also proven to reduce the AHI; however, like PAP, it does not completely overcome adherence challenges. Traditional surgical interventions are further constrained by inconsistent outcomes, risks, and complications related to the procedures, and the potential for recurrence, making them unsuitable for all patient groups.

These challenges underscore the necessity for OSA management to extend beyond the “one-size-fits-all” model of PAP and move toward tailored, multidisciplinary strategies that account for each patient’s specific physiological parameters (airway collapsibility, arousal threshold, loop gain, and pharyngeal muscle responsiveness), anatomical features, polysomnographic profiles, and individual preferences. The incorporation of novel therapies—including HGNS, anti-obesity pharmacotherapies, and medications designed to modulate upper airway muscle tone—in combination with traditional options such as PAP, oral appliances, and surgery, is likely to further evolve OSA management into a more integrated and patient-centered model. These approaches offer the potential to address current therapy limitations and substantially enhance the quality of life for individuals with OSA.

## Figures and Tables

**Table 1:** Comparison of hypoglossal nerve stimulation systems (Inspire, LivaNova, Nyxoah).

	Inspire Medical Systems (Inspire^®^)	LivaNova (aura6000™ System)	Nyxoah (Genio™ System)
FDA approval status (U.S.)	FDA-approved (2014)	Not FDA-approved; PMA submission under review (2024)	FDA-approved (2025)
Mechanism	Unilateral hypoglossal nerve stimulation	Proximal hypoglossal nerve stimulation utilizing six electrodes and adjustable threshold control	Bilateral hypoglossal nerve stimulation
Implantable components	Pulse generator (with battery), respiratory sensing lead, and stimulation cuff electrode	Implantable proximal hypoglossal nerve stimulator (with battery) and associated electrodes	Wireless, battery-free implantable electrodes
External components	Remote control	Remote control	Single-use external patch and activation chip
Quantity and anatomical site of incisions	2–3 incisions (submental, chest, intercostal)	Not comprehensively detailed (potentially 2)	One submental incision
DISE assessment requirement	Required (exclusion: complete concentric collapse at velum)	Indeterminate	Not required
OSA efficacy (AHI reduction)	~20 events/h absolute AHI reduction (~68% relative decrease)	~24 events/h absolute AHI reduction (~54% relative decrease)	~18 events/h absolute AHI reduction (~66% relative decrease)
Most common adverse events	Tongue abrasion, pain, discomfort from electrodes, device malfunction	Lead migration, infection	Temporary localized skin irritation, dysphagia/pain
Estimated cost of device/procedure	~$30,000–40,000	Not specified	~€25,000–30,000 (Europe)
Battery lifespan	~10–11 years	~8–10 years	Permanent (no battery required)
Unique characteristics/advantages	Extensive clinical usage history; based in the U.S.; most commonly utilized	Six electrodes provide high-precision stimulation	Battery-free, offers bilateral stimulation, minimally invasive, does not require DISE, MRI-compatible
Data robustness	Long-term data (>10 years)	Intermediate-term (12 months)	Mainly demonstrates short-term outcomes

FDA, Food and Drug Administration; PMA, premarket approval; DISE, drug-induced sleep endoscopy; AHI, apnea-hypopnea index; ODI, oxygen desaturation index; MRI, magnetic resonance imaging.

**Table 2:** Comparison of GLP-1 receptor agonists (tirzepatide, semaglutide, liraglutide).

	Tirzepatide (Zepbound™, Mounjaro^®^)	Semaglutide (Ozempic^®^, Wegovy^®^, Rybelsus^®^)	Liraglutide (Saxenda^®^)
Mechanism of action	Dual GIP/GLP-1 receptor agonist	GLP-1 receptor agonist	GLP-1 receptor agonist
Route of administration	Once-weekly subcutaneous injection	Weekly subcutaneous injection (Ozempic^®^, Wegovy^®^) or once-daily oral tablet (Rybelsus^®^)	Once-daily subcutaneous injection
FDA approval status	Approved for type 2 diabetes (Mounjaro^®^) and for obesity/OSA (Zepbound™)	Approved for type 2 diabetes (Ozempic^®^, Rybelsus^®^) and for obesity (Wegovy^®^); currently, no prospective clinical trial data in OSA, though efficacy is anticipated to be similar to tirzepatide	Approved for obesity and type 2 diabetes
Weight-loss efficacy	~18–20% mean body weight reduction	~15–17% mean body weight reduction	~5–8% mean body weight reduction
OSA efficacy (AHI reduction)	~25–30 events/h absolute AHI reduction (~55–63% relative decrease)	No prospective OSA clinical trials completed	absolute AHI reduction (~10–25% relative decrease)
Major adverse events	Most common: GI symptoms (diarrhea, nausea, constipation, vomiting, abdominal pain). Rare but serious risks: pancreatitis, gallbladder disease, acute kidney injury, severe allergic reactions, hypoglycemia, and thyroid C-cell tumors.	Common: GI symptoms (nausea, vomiting, diarrhea, constipation, abdominal pain, belching, dyspepsia), typically mild and transient. Rare but serious effects include pancreatitis, gallbladder disease, kidney complications, increased heart rate, and thyroid C-cell tumors (contraindicated in individuals with a personal or family history of MTC or MEN2).	Commonly reported: GI symptoms (nausea, vomiting, diarrhea, constipation). Rare but notable: pancreatitis and gallbladder disease.
Cost and insurance	FDA approval has broadened insurance coverage, now including OSA.	Monthly costs are substantial and can differ based on insurance policies.	Monthly costs are substantial and can differ based on insurance policies.
Additional features	One of the most potent GLP-1 RAs for achieving weight reduction; notably, it is the first FDA-approved medication for OSA, signaling a significant therapeutic advance.	Available in both injectable and oral formulations; extensively utilized for weight management.	Once-daily injectable formulation with a well-established history of clinical application.

GLP-1, glucagon-like peptide-1; GIP, gastric inhibitory polypeptide; FDA, Food and Drug Administration; OSA, obstructive sleep apnea; AHI, apnea-hypopnea index; GI, gastrointestinal; MTC, medullary thyroid carcinoma; MEN2, multiple endocrine neoplasia type 2; RA, receptor agonist.

## Data Availability

Data sharing is not applicable to this study as no datasets were generated or analyzed.

## Abbreviations

The following abbreviations are used in this manuscript:

AHI	Apnea–hypopnea index
BMI	Body mass index
CCC	Complete concentric collapse
CPAP	Continuous positive airway pressure
DISE	Drug-induced sleep endoscopy
ESS	Epworth sleepiness scale
FDA	U.S. Food and Drug Administration
FOSQ	Functional outcomes in sleep questionnaire
GLP-1 RA	Glucagon-like peptide-1 receptor agonist
HGNS	Hypoglossal nerve stimulation
MAD	Mandibular advancement device
MMA	Maxillomandibular advancement
MRI	Magnetic resonance imaging
ODI	Oxygen desaturation index
SA	Obstructive sleep apnea
PAP	Positive airway pressure
SpO_2_	Oxygen saturation
UAS	Upper airway stimulation
UPPP	Uvulopalatopharyngoplasty
